# Clinical challenges and technological breakthroughs in helminthic therapy for diabetes

**DOI:** 10.3389/fimmu.2025.1642707

**Published:** 2025-11-05

**Authors:** Yunhuan Zhu, Xinyi Fei, Ruke Wang, Jiyuan Wang, Xianwei Li, Yijie Zhang, Jialu Xu, Qingzhi Zhao, Keda Chen, Xiaofen Zhang, Hongyu Li

**Affiliations:** ^1^ Key Laboratory of Artificial Organs and Computational Medicine in Zhejiang Province, Shulan International Medical College, Zhejiang Shuren University, Hangzhou, China; ^2^ School of Basic Medicine and Forensic Medicine, Hangzhou Medical College, Hangzhou, China; ^3^ Ocean College, Beibu Gulf University, Qinzhou, China

**Keywords:** helminthic therapy, diabetes mellitus, adverse effects, helminths-derived molecules, risk assessment, clinical monitor

## Abstract

Helminthic therapy, as an emerging strategy for Diabetes Mellitus (DM), demonstrates significant clinical benefits by modulating host immune and metabolic systems. Studies have shown that this approach effectively enhances insulin sensitivity, reduces chronic inflammation, and restores metabolic homeostasis through the regulation of gut microbiota. However, certain diabetic patients undergoing helminthic therapy may encounter risks such as infections or metabolic disturbances, necessitating the development of safer and more precise therapeutic methods. This review, conducted following the PRISMA guidelines, systematically retrieved and analyzed 163 high-quality studies from PubMed, Web of Science, and Scopus databases. It comprehensively evaluates the mechanisms, clinical outcomes, and safety improvement strategies associated with helminthic therapy. To ensure the safe application of this treatment, we propose strategies including genetic editing, real-time monitoring, targeted therapeutics, and helminth-derived molecules, along with a detailed clinical decision-making framework. This framework encompasses the matching of host health status with helminth species selection, guidance on dose optimization and treatment duration, and the application of modern intelligent technologies for real-time monitoring of therapeutic processes and potential adverse effects. Helminthic therapy has demonstrated success in alleviating hyperglycemia, chronic inflammation, and insulin resistance in diabetic patients, offering substantial health benefits through its immunomodulatory and metabolic regulatory effects. These findings suggest that helminthic therapy holds the potential to become a revolutionary approach in the field of DM.

## Highlights

This paper examines the potential of helminthic therapy for DM treatment and the challenges it faces in clinical use. While it has gained attention for its immune-modulating benefits, studies show that diabetic patients may experience more severe side effects. The article reviews these adverse effects and how parasitic infections might speed up DM progression. It also suggests strategies to improve safety, including selecting safer parasites, using gene-editing to reduce parasite virulence, applying real-time monitoring, and developing treatments that target glucose metabolism. These innovations aim to reduce risks and improve the effectiveness of helminthic therapy. The paper further emphasizes that ethical and regulatory safeguards comparable to live biotherapeutics are prerequisites for clinical translation, with helminth-derived molecules excretory/secretory products (ESPs) offering safer and more controllable alternatives.

## 1 Introduction

**Figure 1:**
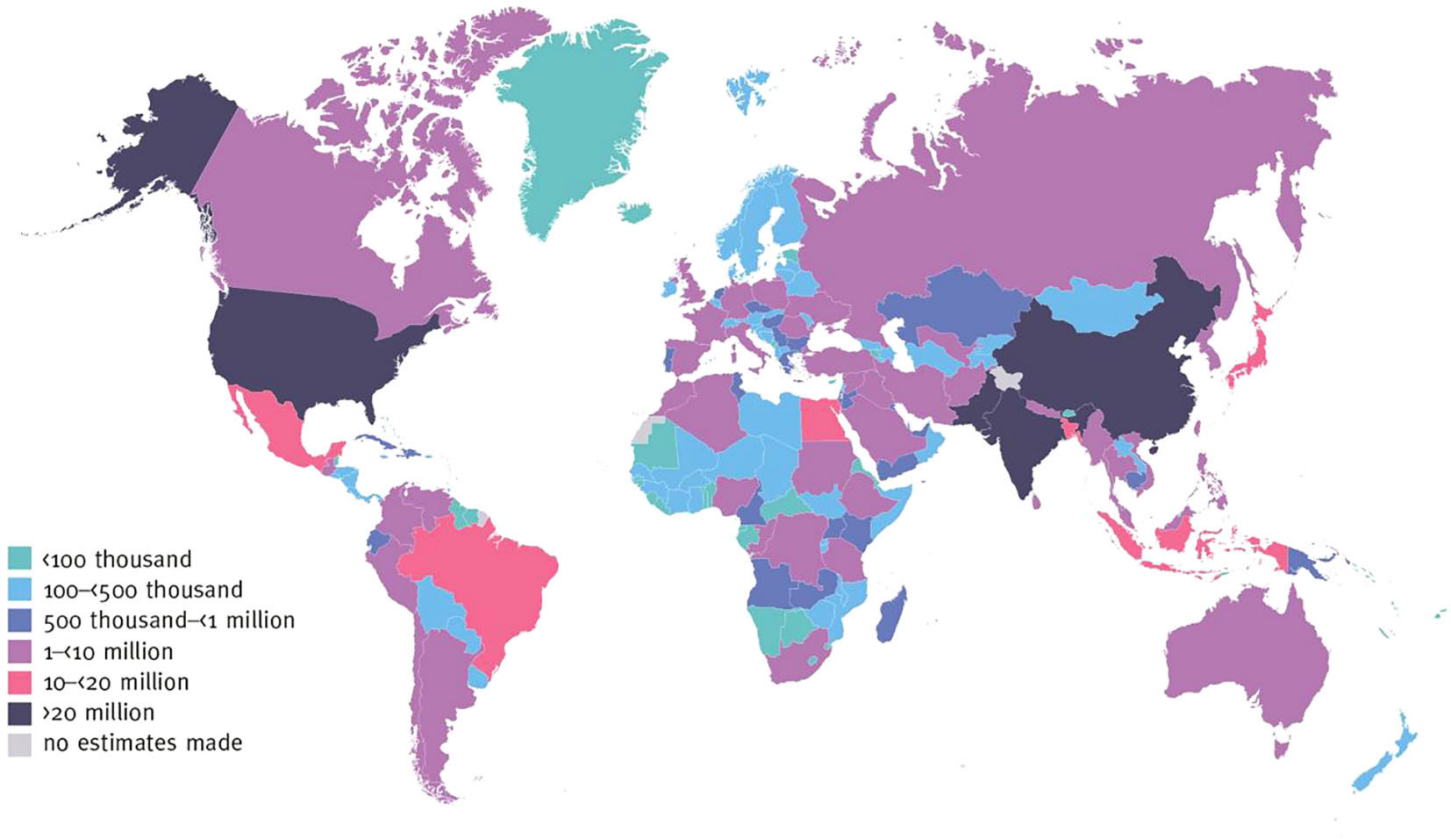
Number of adults with diabetes aged 20–79 years, 2021. International Diabetes Federation.IDF Diabetes Atlas, 10th edn. Brussels, Belgium: 2021. Available at: https://www.diabetesatlas.org.

The global prevalence of Diabetes Mellitus (DM) is alarming, particularly in countries such as China, India, and the United States, where the number of patients has surpassed 20 million (1). In other developing regions, the incidence of DM is also rising rapidly (**Figure 1**). This highlights the urgent need for innovative treatments to address DM as a global health crisis. In this context, helminthic therapy, as a potential treatment strategy for DM, has garnered significant attention due to its unique immunomodulatory properties.

Helminthic therapy involves introducing live parasites to exploit the complex immunological interactions between the parasites and the host (2), aiming to modulate the host’s immune response to counteract the chronic inflammation and metabolic dysregulation associated with DM. A summary of the effects of different parasite species on DM is provided in **Table 1**. Several murine model studies have demonstrated this immunomodulatory capacity, showing that helminth infection can attenuate excessive immune responses and improve insulin resistance (2, 11). Recent clinical trials have also indicated the potential benefits of helminthic therapy, suggesting that parasite-induced immune responses may contribute to restoring immune homeostasis, thereby aiding in blood glucose regulation (3). However, the safety of helminthic therapy in diabetic patients remains a significant concern, as these individuals are particularly vulnerable to infections and metabolic disturbances. Studies have shown that inappropriate parasite selection or excessive dosing can result in serious complications, including disruptions to the gut microbiome and pancreatic function (12, 13). To mitigate these risks, several innovative strategies are critical. These include the selection of less pathogenic helminths, the use of genetic modifications to reduce pathogenicity, and the implementation of real-time biomarker analysis to monitor therapeutic outcomes (7, 14). Additionally, the development of anti-parasitic drugs that work synergistically with helminthic therapy, particularly those targeting glucose metabolism, could further enhance the safety and effectiveness of this approach. Not only live helminth but also helminth-derived products are being used as therapeutics including in DM. Numerous studies have demonstrated that parasite-associated molecules exhibit regulatory effects on the host, akin to those observed *in vivo*, and these findings have been comprehensively summarized in various reviews (15–17). Furthermore, based on our analysis and synthesis of high-quality literature, we have initially summarized key aspects of risk assessment, dosage adjustment, and precautions in the clinical application of helminth therapy. By employing these strategies, the safety of helminthic therapy can be better assured, facilitating its potential application in the treatment of DM and other chronic diseases.

**Table 1:** Parasitic species and their potential therapeutic effects on diabetes.

Parasitic species	Key effects on diabetes	Mechanisms	Associated risks	Conflicting evidence	Ref.
*N. americanus*	Improves insulin sensitivity; reduces inflammation; alters gut microbiota	Induction of Tregs; secretion of anti-inflammatory proteins	Anemia; gastrointestinal symptoms	Early-phase human studies in MS/CeD report safe/tolerated exposure	(3, 4)
*TSO*	Reduces pancreatic inflammation; improves insulin resistance; transient effects	Activation of anti-inflammatory cytokines (IL-10); M2 macrophage induction	Requires repeated doses; variable efficacy	Safety profile varies by indication: safe in Crohn’s phase I/II	(5)
*S. mansoni*	Reduces diabetes and metabolic syndrome risk; improves lipid profiles	Secretion of omega-1 glycoprotein; Th2 immune response	Hepatic fibrosis in chronic infections	Egg deposition in the liver may cause granulomatous hepatobiliary involvement; metabolic readouts can be confounded by infection stage and burden	(6)
*S. stercoralis*	Enhances insulin sensitivity; reduces systemic inflammation; alters leptin-to-adiponectin ratio	Suppresses pro-inflammatory cytokines; improves gut microbiota diversity	Hyperinfection syndrome in immunocompromised hosts	Signals of improved insulin sensitivity in some cohorts; hyperinfection syndrome reported in diabetic patients and co-infections	(7, 8)
*T. gondii*	Mixed effects: worsens insulin resistance in some cases; may protect against obesity-related metabolic syndrome	Pro-inflammatory cytokines (IL-12, IFN-γ); modulation of immune pathways	Risk of cerebral toxoplasmosis	Th1/Th17-linked inflammation; β-cell injury; worsened insulin resistance (stage/burden dependent).	(9)
*A. duodenale*	Improves glucose metabolism; reduces inflammation	Similar to *N. americanus*	Higher risk of anemia	anemia/protein loss and GI adverse events; public-health burden in endemic settings.	(10)

TSO, Trichuris suis Ova; A. duodenale, Ancylostoma duodenale.

Helminths are the focus; protozoan data are shown as comparative context.

## 2 Methods

### 2.1 Search strategy

This study utilized a systematic review methodology, adhering to the PRISMA (Preferred Reporting Items for Systematic Reviews and Meta-Analyses) guidelines for literature screening and inclusion. The literature search was conducted up to January 2025, with data sources including PubMed, Web of Science, Scopus. The search strategy incorporated both keywords and subject terms, focusing on parasites (Parasite*, Helminth*, Parasitic therapy, Helminthic therapy, Worm therapy), diabetes (Diabetes, Type 1 diabetes, Type 2 diabetes, Diabetes mellitus), and related immunological and metabolic indicators (e.g., Immune, Inflammation, Cytokines, Insulin sensitivity, Glucose metabolism, homeostatic model assessment of insulin resistance(HOMA-IR), Gut microbiota, Adverse effects, Toxicity, Infection risk, Monitoring, Surveillance, Biomarkers, Diagnostics, Detection, Targets, Molecules, Proteins, Assessment, Evaluation). The search terms were combined as follows: [(Parasite*) OR (Helminth*) OR (Parasitic therapy) OR (Helminthic therapy) OR (Worm therapy)] AND [(diabetes) OR (Type 1 diabetes) OR (Type 2 diabetes) OR (diabetes mellitus)] AND [(immune) OR (inflammation) OR (cytokines) OR (insulin sensitivity) OR (glucose metabolism) OR (HOMA-IR) OR (gut microbiota) OR (adverse) OR (toxicity) OR (infection risk) OR (Monitoring) OR (Surveillance) OR (Biomarkers) OR (Diagnostics) OR (Detection) OR (Targets) OR (Molecules) OR (Proteins) OR (Assessment) OR (Evaluation)]. Additionally, citation searching and manual screening of references from relevant review articles were performed to identify further studies that met the inclusion criteria.

### 2.2 Eligibility criteria

The inclusion criteria for this study were as follows: (*i*) Research examining the effects of parasitic infections or helminthic therapy on DM (Type 1 or Type 2); (*ii*) Studies investigating biological mechanisms, including immune responses, inflammation, cytokine levels, insulin sensitivity, or glucose metabolism; (*iii*) Original research designs such as randomized controlled trials (RCTs), cohort studies, or case-control studies; (*iv*) Articles published in English; (*v*) Studies providing complete experimental data, clearly defined methodologies, and comprehensive results. The exclusion criteria included: (*i*) Articles unrelated to the research topic, such as those not addressing DM or parasitic infections; (*ii*) Non-original research, including review articles, conference abstracts, opinion pieces, or case reports; (*iii*) Studies limited to animal experiments without clinical or human data; (*iv*) Non-English publications without available translations; (*v*) Research with outdated findings that no longer align with the objectives of this study.

### 2.3 Screening results

**Figure 2:**
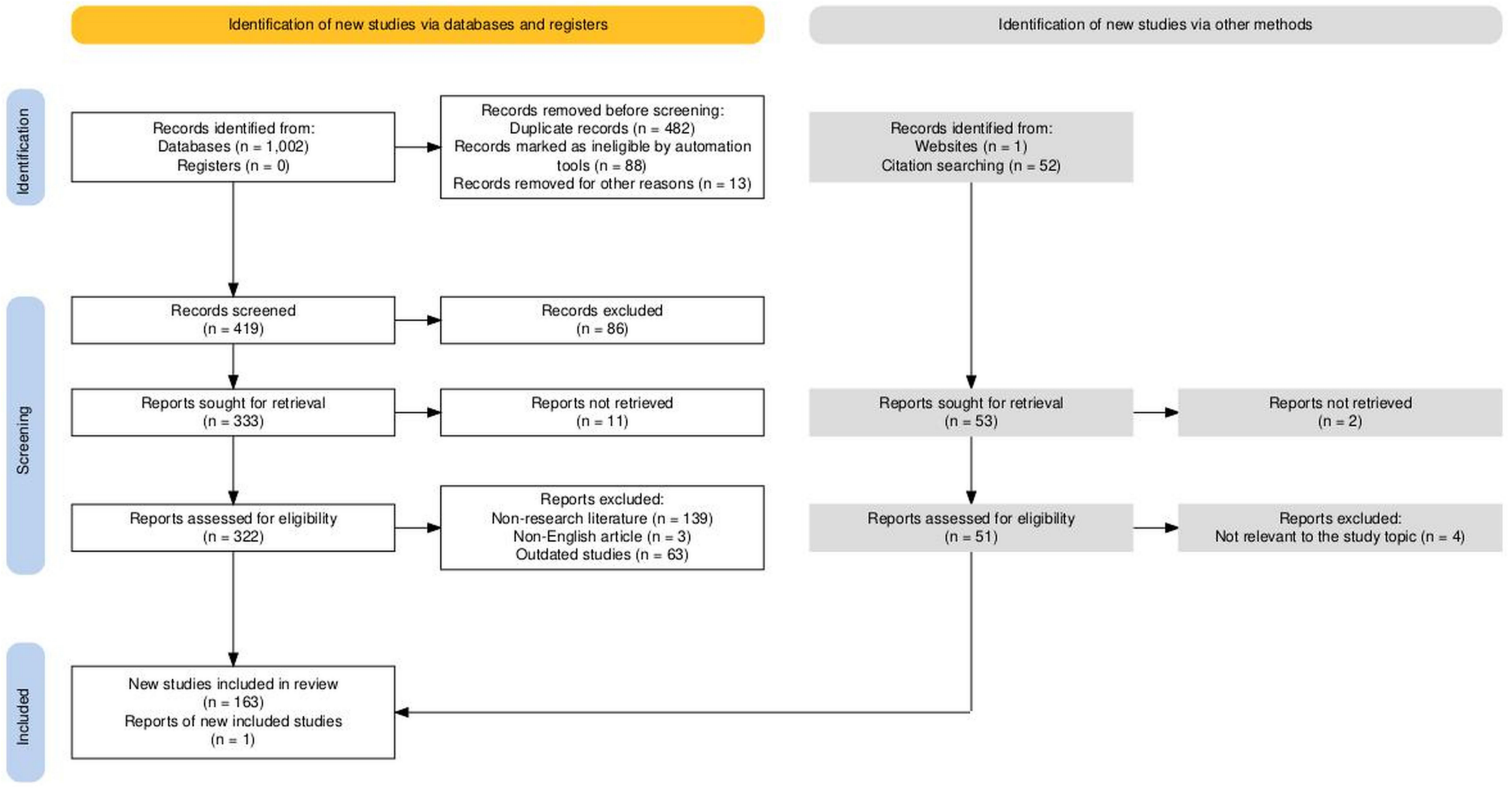
PRISMA flowchart for literature search. Haddaway, N. R., Page, M. J., Pritchard, C. C., & McGuinness, L. A. (2022). PRISMA2020: An R package and Shiny app for producing PRISMA 2020-compliant flow diagrams, with interactivity for optimized digital transparency and Open Synthesis Campbell Systematic Reviews, 18, e1230. https://doi.org/10.1002/cl2.1230.

This study followed the PRISMA framework for literature screening (**Figure 2**). Two authors independently conducted database searches, initially retrieving 1,002 articles. After removing 482 duplicates, an automated screening tool (EndnoteX20) excluded 88 articles unrelated to the research topic. Additionally, 13 articles were removed for being outside the scope of the study, leaving 419 articles for initial screening. Titles and abstracts were reviewed independently by both authors, resulting in the exclusion of 86 irrelevant studies. This process left 333 articles for full-text review. Of these, 11 articles could not be accessed and were excluded. A detailed eligibility assessment was then carried out independently on 322 articles, leading to the removal of 139 non-research articles, 3 non-English studies, and 63 studies excluded due to outdated findings or insufficient methodologies. Citation tracking, performed independently by each author, identified 53 potentially relevant articles. Of these, 2 articles were excluded due to access issues, and 4 unrelated studies were removed after further evaluation. Ultimately, 163 studies meeting the inclusion criteria were included, along with 1 newly published research report.

## 3 The mechanisms of helminthic therapy

### 3.1 Helminths affect host metabolism

Helminths remodel host metabolism through two interconnected routes. A direct route targets epithelial and neuro-endocrine pathways—driven by IL-4/IL-13/STAT6 signaling and helminth excretory/secretory products (ESPs)—to reprogram glucose transport and local inflammation. An indirect route operates via the gut microbiota, increasing short-chain fatty acids (SCFAs) and barrier integrity. The following Sections 3.1.1 (direct) and 3.1.2 (microbiota-mediated) detail these complementary mechanisms.

#### 3.1.1 Direct metabolic modulation by helminths

Helminth infection engages IL-4/IL-13–STAT6 signaling to tune intestinal epithelial glucose transport, thereby rebalancing luminal handling and dampening nutrient-driven inflammation.

Helminth infections significantly impact host energy metabolism by altering glucose absorption and metabolism. Initial research has demonstrated that these changes may occur through modifications in intestinal glucose transport receptors or Ach-induced contraction responses, mediated by IL-4 or IL-13 stimulated signal transducer and activator of STAT6 signaling within the enteric nervous system (18, 19). It has been shown in subsequent studies that helminths also modify intestinal M2 cells, decreasing sodium-glucose cotransporter 1 (SGLT1) expression while concurrently downregulating GLUT2 and upregulating GLUT1, thus shifting glucose absorption pathways (20, 21). *In vivo*, experiments by Koehler et al. in mice have demonstrated significant transcriptional reductions in GLUT1, STAT6, hypoxia-inducible factor 1-alpha (HIF-1α), and peptide transporter 1 (PepT1) in the jejunum, and in GLUT2 and PepT1 expression in the ileum, indicating a detrimental effect on physiological transport mechanisms (22).

Helminth excretory/secretory products (ESPs) act directly on epithelial transport and local cytokine milieus (hereafter “ESPs”). Recent findings have revealed the suppression of the transporter protein GLUT8 during *Opisthorchis viverrini (O. viverrini)* infections and alterations in glucose metabolism around host cells due to the total excretory-secretory (ES) antigens of *Ascaris suum (A. suum)* (23, 24). Functionally, ESP-triggered signaling yields rapid, microbiota-independent improvements in epithelial glucose handling and local inflammatory tone.

Direct reinforcement of mucosal/tissue repair programs and alternatively activated M2 macrophages reduces contact-dependent inflammation and supports metabolic recovery. Adult *Schistosoma mansoni (S. mansoni)* worms reside in host veins, and their eggs accumulate in the liver, affecting both adipose and liver metabolism (2). Cytokines such as IL-4 and IL-13 are critical for adipose tissue homeostasis (25). In obese mice, the injection of *S. mansoni* antigens has been found to stimulate IL-33 release from adipocytes, activating group 2 innate lymphoid cells (ILC2s) and promoting the infiltration of M2 cells and eosinophils into white adipose tissue, thereby enhancing metabolic functions (26). Additionally, Ni et al. discovered that *Schistosoma japonicum (S. japonicum)* infections regulate lipid metabolism via miRNAs pathways: the interaction of Sjp40 with CD36 on hepatocytes inhibits miR-802, enhancing the expression of Prkab1 or Prkaa1 and increasing phosphorylated AMP-activated protein kinase (AMPK) levels, leading to reduced hepatic lipid synthesis (27). Notably, *Heligmosomoides polygyrus (H. polygyrus)* infection in mice fed a high-fat diet (HFD) has been shown to prevent obesity, dyslipidemia, and glucose intolerance (28). These studies illuminate the complex interactions between parasitic infections and host metabolic processes, offering innovative approaches for managing metabolic disorders. By stabilizing local inflammatory tone and hepatic/lipid pathways, these direct mechanisms help lower peripheral and hepatic insulin resistance and consolidate early glycemic benefits.

Taken together, these direct epithelial and neuro-endocrine mechanisms initiate early improvements in glucose handling that are further amplified by microbiota-mediated processes (Section 3.1.2).

#### 3.1.2 Microbiota-mediated metabolic effects

Since the initial germ-free mouse experiments, the role of the gut microbiota in regulating energy metabolism has been extensively studied (29). It has been established that microbial populations are crucial for energy extraction, lipid storage, and vitamin synthesis. Additionally, infections with helminths such as *H. polygyrus*, *Nippostrongylus brasiliensis (N. brasiliensis)*, and *Trichuris muris (T. muris)*, have been shown to increase the population of Lactobacillaceae, recognized for its probiotic potential through immune response regulation (30, 31). Research has found that parasites depend on the host’s gut microbiota for nutrient acquisition and survival, suggesting that helminths may modulate the host’s metabolic state by altering the gut microbiome (32).

Further investigations have explored the interactions between parasites and the host’s gut bacterial communities, particularly noting the diversity of Blastocystis sp (33)., which is associated with an increased risk of type 2 diabetes (T2D), IR, and metabolic syndrome (MetS). It has been shown that parasites secrete substances that inhibit specific bacterial populations while promoting the production of short-chain fatty acids (SCFAs) (34), such as butyrate, propionate, and acetate, by the gut microbiome. Butyrate and propionate are particularly noted for enhancing insulin sensitivity in muscle and fat tissues, reducing blood glucose levels, and aiding obesity prevention and T2D risk reduction (35–38). Significant reductions in weight gain under a high-fat diet in mother rats infected with *H. polygyrus* and their offspring have been attributed to changes in the gut microbiome and elevated SCFA levels (39).

Microbiota-dependent strengthening of tight junctions and immunoglobulin A (IgA)-mediated containment reduces endotoxin leakage and systemic inflammatory tone. SCFAs also play a positive role by strengthening the intestinal barrier, reducing inflammation and metabolic endotoxemia, thereby mitigating systemic inflammation and IR in diabetics (40). Recent research has linked improvements in insulin sensitivity in diet-induced obesity (DIO) mice and Venezuelan hookworm infections to microbial community alterations, notably an increase in lactobacilli and decreased intestinal permeability. These microbiota alterations enhance host metabolic functions by increasing levels of anti-inflammatory cytokines, transitioning adipose tissue MΦ from an M1 to an M2 phenotype, boosting the expression of tight junction proteins in intestinal cells, and lowering serum lipopolysaccharides (LPS) (41). Consequently, diminished microbial translocation lowers systemic TNF-α/IL-6 and supports restoration of insulin signaling in metabolic tissues. Experimental evidence indicates that increases in SCFAs in helminth-infected mice strongly correlate with shifts in microbial populations. Studies have observed a rise in the Clostridiales order, known for its efficient SCFA production, across various infection models, highlighting the potential of SCFAs in modifying obesity and enhancing insulin sensitivity (35, 42–45).

Notably, associations with Blastocystis sp. remain heterogeneous; we treat these findings as comparative context rather than core evidence for helminthic therapy. Further investigations have explored the interactions between parasites and the host’s gut bacterial communities, particularly noting the diversity of Blastocystis sp (33)., which is associated with an increased risk of type 2 diabetes (T2D), insulin resistance(IR), and metabolic syndrome (MetS). Accordingly, non-helminth species are summarized in **Table 1** and discussed in Section 4.2 to explain apparent contradictions without conflating pathogen classes.

Further studies indicate that *H. polygyrus* infection may offer protective effects against obesity by indirectly modulating norepinephrine (NE) concentrations through microbial community changes (46), with Th2 cells significantly facilitating metabolic enhancements by influencing microbial shifts (47).

### 3.2 Helminths regulate host immune responses

**Figure 3:**
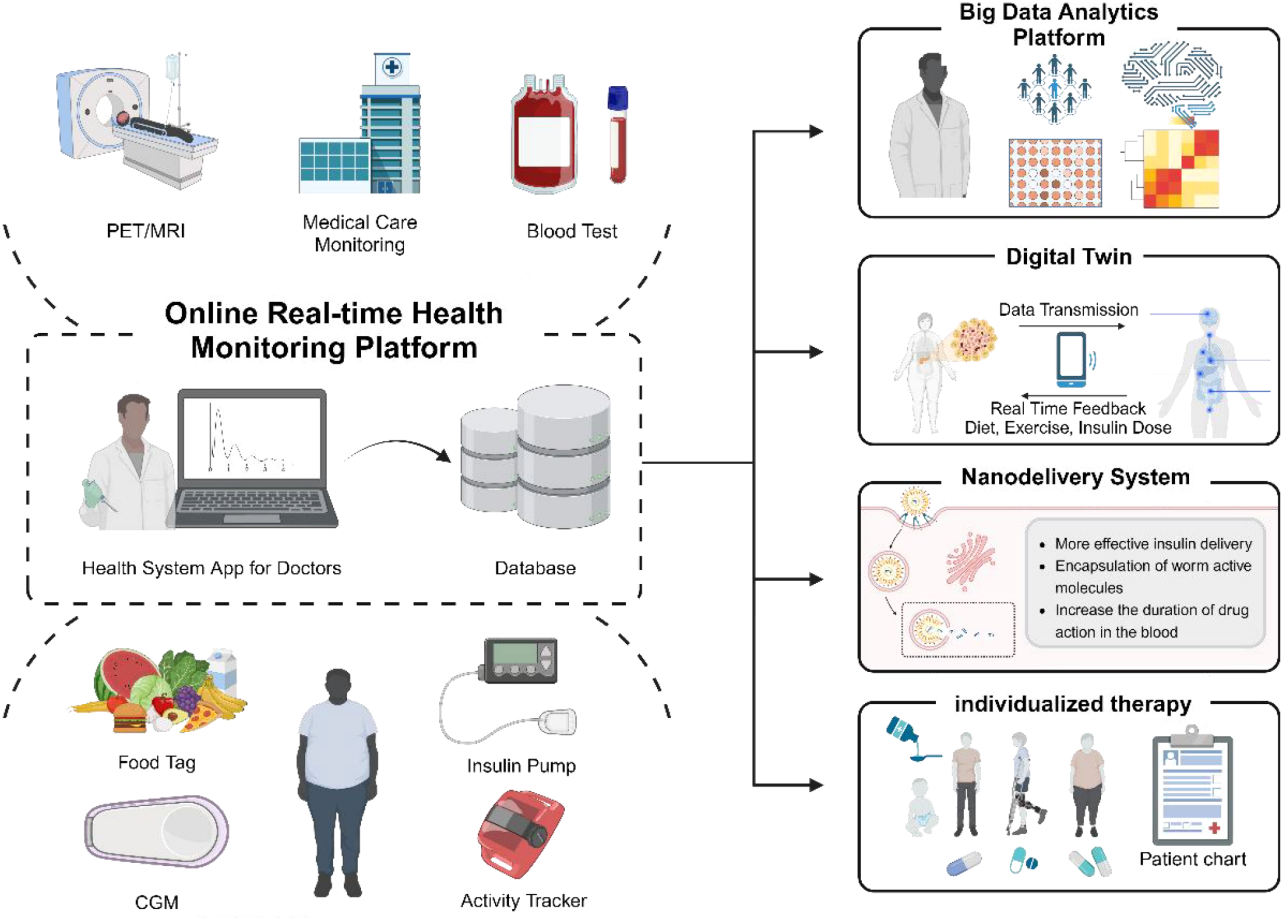
Anticipated future health monitoring platform used for treating T2D with parasitic therapy. This platform seamlessly integrates a range of data sources, including portable medical devices, nutritional tracking, and monitoring of physiological parameters, facilitating the real-time upload of health information to a cloud-based database. Leveraging big data analytics and digital twin technology, it can accurately simulate and dynamically adjust to individual health conditions based on the acquired data. Additionally, applying nano-delivery technology, particularly encapsulating active molecules derived from parasites, significantly improves medications’ bioavailability and circulation duration. By synthesizing these advanced technologies, the platform crafts personalized treatment plans that enhance the therapeutic outcomes for patients.

Many parasites must deploy evasion strategies against the mammalian immune system to ensure long-term survival within their definitive hosts. These parasites can cause chronic infections in host tissues and typically induce a shift in the host immune response toward an anti-inflammatory phenotype while suppressing pro-inflammatory responses (**Figure 3**) (48).

Inflammation triggered by macrophages (MΦ) plays a crucial role in the dysfunction of pancreatic β-cells. Within the islets, MΦ regulates the survival and metabolic activity of β-cells by acting as sensors, engaging in bi-directional exchanges of signals such as cytokines, hormones, and growth factors (49). However, in the progression of T2D, inflammatory signals disrupt this physiological equilibrium, resulting in interactions between MΦ and β-cells that lead to β-cell dysfunction and apoptosis. Specifically, early-stage T2D is characterized by pancreatic hypoxia that induces cellular stress and necrosis, releasing fatty acids and intracellular substances that activate the transcription inhibitor basic helix-loop-helix family member e40 (BHLHE40) (50), impairing insulin secretion. These conditions foster the transformation of MΦ into a pro-inflammatory state (M1 type), with increased levels of IL-12 and decreased IL-10 exacerbating the inflammation (51, 52). Furthermore, the upregulation of miR-212-5p in M1 macrophage-derived exosomes (M1-Exos) and high-fat diet-derived exosomes (HFD-Exos) influences β-cell insulin secretion by modulating the sirtuin two gene and the Akt/GSK-3β/β-catenin pathway (53), also generating pro-inflammatory mediators such as nitric oxide (NO) and reactive oxygen species (ROS), and expressing chemokine receptors like C-C motif CCR5, C-X-C motif CXCR3, and CCR8, which recruit pro-inflammatory cells (54). Parasitic infections often induce a Th2 immune response in the host, promoting intestinal “clearance and excretion” processes like increased mucus production and intestinal contractions, thus altering the gut microbiota (43, 55). Mishra et al. observed that in obese mice infected with *S. mansoni*, MΦ differentiation in white adipose tissue shifted towards an anti-inflammatory M2 phenotype, benefiting reductions in body weight and fat mass (FM) and enhancing glucose tolerance and insulin sensitivity (56). This was further supported by a study by Kang, where mice on a high-fat diet (HFD) infected with whipworms showed significantly lower increases in body weight, fat content, total cholesterol, and food efficiency ratio levels compared to uninfected controls (57). This protective effect is closely associated with the parasite’s ability to synergistically induce M2 through anti-inflammatory mediators such as IL-4 (58), promoting their proliferation and inhibiting inflammatory responses.

Numerous studies have confirmed that several worms can modulate human T helper 1 (Th1)/T helper 2 (Th2) cells and cytokines, steering inflammatory responses towards a Th2-dominated immune reaction and ameliorating conditions like obesity or malnutrition (59). Research involving subjects infected with worms has shown varying improvements in metabolic health. Typically, worm infections increase Th2 cytokines, such as IL-4 and IL-13, while decreasing Th1 cytokines like IL-12 and IFN-γ (60). These cytokines further orchestrate the immune response, facilitating the recruitment of eosinophils, the production of B-cell-like entities, and the activation of alternative MΦ (61, 62). The equilibrium between T helper 17 (Th17) cells and Treg cells, both critical T cell subsets, is essential for maintaining immune homeostasis. Clinical data reveal that diabetic patients exhibit significantly elevated levels of Th17 and Th1 cells and reduced Treg cells (P < 0.01) (63). Furthermore, regulatory T cells(Treg) levels inversely correlate with high glucose levels, and diabetic patients experience more significant fluctuations in average glucose levels compared to those with normal albuminuria (P < 0.05) (64). It is hypothesized that elevated blood glucose may increase Th1 and Th17 cells and inflammatory cytokines, impeding improvements in T2D (65). Experimental evidence suggests that infection with the *H. polygyrus* parasite increases the expression of IL-10 and adiponectin, reduces levels of leptin and anti-insulin, and enhances the infiltration of Th2 cells and eosinophils in adipose tissue—particularly enhancing the functionality and expression of activation markers, such as latency-associated peptide and CD134 in Treg cells (66).

The proliferation of eosinophils (EOS) is a distinguishing feature of host immune responses, enabling the differentiation of parasitic infections from other pathogenic challenges. In T2D development, eosinophils are pivotal in mediating Th2-type immune responses. IL-10, secreted by eosinophils, orchestrates these responses and limits local NO production. Parasites dependent on chronic infections may leverage this adaptive mechanism to sustain their presence and manipulate host immune responses (67, 68). Research indicates that eosinophils are the primary cells expressing IL-4 in the adipose tissue of mice. When subjected to an HFD, mice lacking eosinophils exhibit increased adiposity, diminished glucose tolerance, and heightened IR. Therefore, eosinophils are essential for maintaining glucose equilibrium and countering metabolic disturbances induced by HFD (69). In experimental models of diet-induced T2D in mice, administering the parasitic nematode *N. brasiliensis* for prevention and treatment significantly ameliorated fasting glucose levels, oral glucose tolerance, and weight gain, a process linked to increased EOS counts in mesenteric lymph nodes, liver, and adipose tissue (70).

Additionally, Mast cells (MCs) have been shown to influence immune regulation significantly. During infections with *Hymenolepis diminuta (H. diminuta)*, activated MCs are instrumental in modulating the production of Th2 cytokines, contributing to a robust Th2-skewed immune response and expedited recovery in mice (71, 72).

### 3.3 Clinical researches

To balance mechanistic insights with human evidence, we synthesize observational and interventional data linking helminth exposure to metabolic outcomes, then summarize current trial status and the outstanding gaps that should guide subsequent Phase II/III designs.

Observational cohorts. Population studies in helminth-endemic settings suggest a protective association with metabolic traits. In Flores Island, Indonesia, soil-transmitted helminth (STH) infection correlated with lower insulin resistance (HOMA-IR), with a dose-response pattern whereby each additional STH species was associated with further HOMA-IR reduction (73). In rural China, previous schistosome infection was linked to lower fasting and postprandial glucose, lower HbA1c and HOMA-IR, and lower prevalence of diabetes and metabolic syndrome (adjusted OR for diabetes 0.51; 95% CI 0.34–0.77) (74). In Northern Australia, Strongyloides stercoralis seropositivity showed an inverse association with type 2 diabetes in Aboriginal adults, reinforcing the epidemiological signal across distinct host and parasite contexts (75). Collectively, these cohorts indicate that helminth exposure may align with improved glycemic indices, while acknowledging residual confounding and the cross-sectional nature of many datasets.

Early-phase trials. The first randomized, double-blind, placebo-controlled Phase Ib trial of *Necator americanus* in adults at risk of type 2 diabetes (Australia; n=40) prioritized safety and assessed metabolic secondary outcomes: participants inoculated with 20 or 40 L3 larvae showed lower HOMA-IR and fasting glucose at 12 months, with body mass reduction observed in the 20-larvae group at 24 months; adverse events were mainly mild-to-moderate gastrointestinal symptoms, and completion rates were comparable to placebo (76). The published trial protocol details dose, follow-up (24 months), and predefined metabolic endpoints (HOMA-IR, body composition) (3). By contrast, multiple RCTs of Trichuris suis ova (TSO) in non-metabolic indications (e.g., allergic rhinitis, Crohn’s disease) demonstrate acceptable safety but inconsistent efficacy, underscoring feasibility while highlighting the need for indication-specific endpoints and adequately powered designs before translation to diabetes care (77, 78).

Limitations and next steps. Despite encouraging signals, heterogeneity in host background, parasite species/dose, endpoints (HOMA-IR vs OGTT vs HbA1c), and follow-up limits inference. Importantly, a cluster-randomized trial in Indonesia showed community-level deworming did not change insulin resistance overall but significantly increased insulin resistance in the subgroup with microscopy-confirmed helminth infection, suggesting reverse-causality-consistent effects and emphasizing the value of stratification and baseline phenotyping in future trials (79). Mechanistic analyses from the same program linked deworming to adipokine shifts (↑ leptin/adiponectin ratio) that may partially mediate metabolic changes (80). Future studies should power for standardized metabolic endpoints (HOMA-IR, HbA1c, OGTT), prespecify safety monitoring (including anemia and GI AEs), and consider comparators such as helminth-derived molecules/ESPs to mitigate risks while preserving immunometabolic benefits.

**Figure 4:**
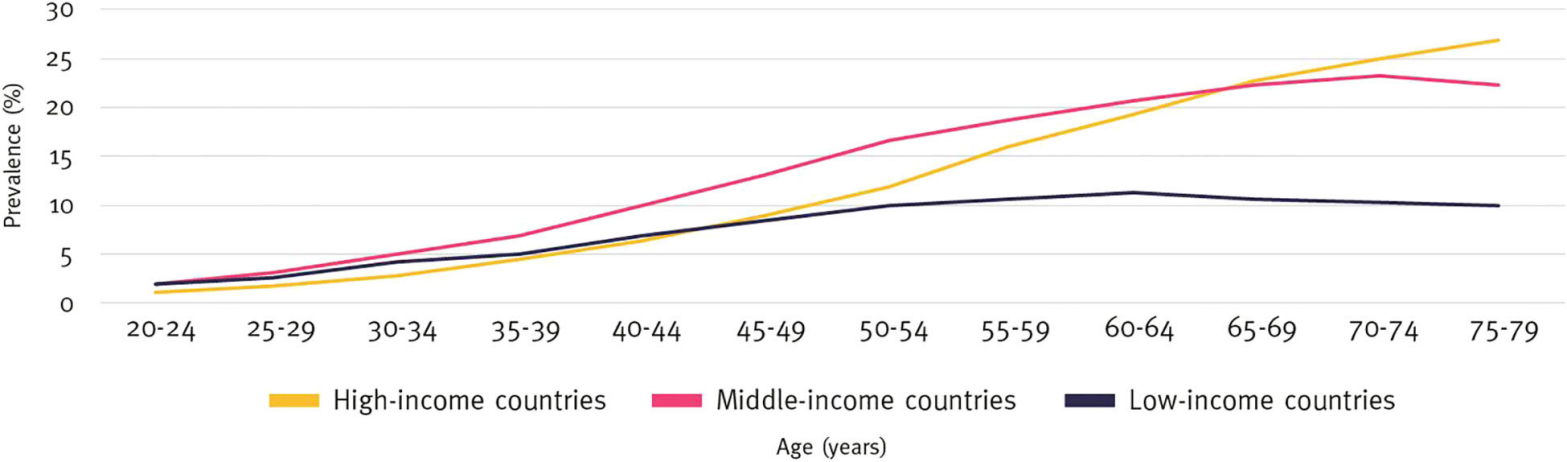
Prevalence of diabetes by age and income group (%), 2021. International Diabetes Federation.IDF Diabetes Atlas, 10th edn. Brussels, Belgium: 2021. Available at: https://www.diabetesatlas.org.

Studies have observed an inverse relationship between parasitic infections and metabolic diseases such as DM and atherosclerosis. For example, the prevalence of metabolic syndrome was significantly lower (18.28%) in individuals with schistosome infections compared to those without (34.01%) (17, 81). This aligns with the “hygiene hypothesis,” which associates lower DM incidence in low-income countries, where parasitic infections are more common, with reduced immune-mediated metabolic disorders (74, 82–84) (**Figure 4**).

Subsequent studies have demonstrated that helminth infections may protect against type 2 diabetes (T2D) and metabolic syndrome by modulating metabolic processes. For instance, a randomized controlled trial in Indonesia revealed that participants infected with helminths had an insulin resistance index (HOMA-IR) approximately 16% lower than uninfected individuals; however, this improvement diminished by about 10% following deworming treatment (85). In a large cross-sectional study in rural China, the prevalence of DM was 14.9% among individuals who had previously been infected with schistosomes, compared to 25.4% among those who had never been infected. This study also reported that the prevalence of metabolic syndrome was 14.0% in those with a history of infection, compared to 35.0% in those without (74). Similarly, an Australian study observed that the incidence of T2D was about 13% lower in individuals infected with *Strongyloides stercoralis (S. stercoralis)* compared to uninfected individuals, with the risk of DM increasing by approximately 10% after deworming treatment (86). A randomized controlled trial in Uganda indicated that individuals infected with *S. mansoni* had low-density lipoprotein cholesterol (LDL-c) levels approximately 0.26 mmol/L lower than uninfected individuals (2.37 mmol/L vs. 2.63 mmol/L), which could potentially lower their cardiovascular disease risk (87). These findings suggest that helminth infections play a protective role against T2D and metabolic syndrome through intricate metabolic mechanisms. Research by Pierce et al. found that low-dose hookworm infections are safe for managing inflammatory diseases and obesity-related metabolic syndrome (3, 88–90). Although these studies did not directly assess metabolic health, hookworm infections have been shown to elicit immune responses (91) and alter the gut microbiome (92), which might be advantageous for T2D. An Australian study further examined the effects of hookworms on T2D risk, where 40 participants were randomly assigned to receive either a placebo or varying doses of hookworm larvae. After two inoculations, participants were evaluated every six months over two years. The study primarily assessed safety and changes in insulin resistance. Results indicated that, although adverse events were more frequent in the hookworm group, the completion rate was similar, and this group exhibited improvements in fasting blood glucose and insulin resistance after one year, with a reduction in body weight observed after two years. These findings suggest that hookworm infection may be a safe and effective intervention for individuals at risk for T2D (76). Helminth infections have also been shown to significantly impact fat metabolism-related hormones. Studies indicate that leptin levels decrease significantly in infected individuals, while adiponectin levels increase, leading to a reduced leptin-to-adiponectin ratio (LAR). This hormonal adjustment enhances insulin sensitivity, further supporting the beneficial effects of helminth infections on metabolic health (93, 94). Additionally, helminth infections may affect the progression of T2D by inhibiting angiogenic pathways. For example, during infection, serum levels of vascular endothelial growth factor (VEGF) significantly decrease, with levels returning to baseline following deworming treatment. This suggests that helminth infections might modulate DM progression by inhibiting angiogenesis (95).

Patients with hypoglycemia and complicated T2D exhibit significantly elevated levels of total white blood cells, neutrophils, monocytes, the neutrophil-to-lymphocyte ratio (NLR), the monocyte-to-lymphocyte ratio (MLR), the mean platelet volume-to-lymphocyte ratio (MPVLR), and the platelet-to-lymphocyte ratio (PLR). In contrast, red blood cell parameters and indices are significantly decreased in cases of hypoglycemia and complicated T2D (96). Consequently, the relationship between parasitic infections and T2D can also be understood through immune regulation mechanisms. Multiple studies have demonstrated that infection with *S. stercoralis* can significantly reduce the levels of pro-inflammatory cytokines in patients with T2D, including key inflammatory mediators such as IL-1β, IL-6, and TNF-α. This inhibitory effect diminishes following antiparasitic treatment, with cytokine levels returning to near-normal levels comparable to uninfected individuals, underscoring the crucial role of helminth infections in modulating inflammatory responses (97). Furthermore, hookworm infections have been shown to alter the composition of the gut microbiota, thereby influencing the host’s immune response. Following infection, there is a significant increase in the proportion of anaerobic bacteria such as *Lactobacillus* and *Bifidobacterium* in the gut. This increase is closely associated with reduced inflammation and improved insulin sensitivity (98). Continuing research into 2024 has further examined the effects of *S. stercoralis* infection on the complement system, a key component of immune regulation. The findings indicate that in T2D patients infected with *S. stercoralis*, the levels of complement proteins [(e.g., C1q, C4b, and MBL (lectin))] and complement regulatory factors (e.g., factor B and factor D) partially recover after antiparasitic treatment, suggesting that *S. stercoralis* may reduce inflammation by modulating complement activation (7). Taken together, helminth infections appear to offer significant protective effects against metabolic diseases such as T2D through mechanisms that include the inhibition of inflammation, regulation of lipid metabolism, modulation of angiogenesis, and enhancement of gut microbiota composition.

## 4 Adverse effects of helminthic therapy

### 4.1 Patients with diabetes exhibit an increased susceptibility to infections by parasites

Currently, parasites are widely recognized as a significant pathogenic factor associated with various health issues, including malnutrition (99), anemia (100), and intestinal obstruction (101). However, their connection with DM is not universal. Salvador, for example, identified no correlation between trichostrongyliasis and DM or other metabolic disorders (102).

Meta-analytical studies indicate that diabetic patients have a higher overall prevalence of intestinal parasitic infections (IPIs) than healthy controls, with rates of 25.7% compared to 15.5%. Infections by Cryptosporidium, Entamoeba histolytica, and hookworms are notably more common in these patients, with odds ratios (ORs) of 3.30, 1.57, and 6.09, respectively (103). These findings highlight the critical need for targeted health education and preventative strategies for this population. Most experts agree that patients with T2D should be shielded from parasitic infections due to their compromised glucose regulation (104, 105), which could exacerbate susceptibility to infections like those caused by acarids, potentially accelerating parasite growth and enhancing virulence (106, 107). Abdelhamid et al., using glial fibrillary acidic protein (GFAP) as a diagnostic marker, observed that mice resistant to cerebral toxoplasmosis exhibited more severe encephalitis when infected with *Toxoplasma gondii (T. gondii)* post-DM onset (108). Li’s study in China revealed significantly higher seropositivity rates for T. gondii in patients with type 1 diabetes (T1D) and T2D, as well as gestational DM, compared to controls (109). However, a key concern is the potential for exaggerated immune responses in certain individuals. Some parasites can trigger excessive IgE-mediated immune reactions, leading to allergic responses such as asthma or eczema, particularly in genetically susceptible populations (110, 111). Additionally, patients with autoimmune disorders such as rheumatoid arthritis or systemic lupus erythematosus (SLE) may experience worsened disease activity due to helminth-induced immune modulation (112).

Contrarily, not all parasitic infections confer protection against DM; some may trigger its onset. The prevalence of IPIs in such case studies was higher in the affected population than in controls, with a noted correlation (OR, 1.80) (103). Additional meta-analytical research is required to explore the interplay between T1D-induced immunological changes and the risk of T. gondii infection, and vice versa (113–115). Moreover, a case report highlighted the death of a 40-year-old diabetic patient with a history of alcoholism due to severe *S. stercoralis* infection (116). It remains prudent for most individuals, especially those at high risk for DM, to maintain environmental cleanliness and minimize exposure to parasitic infections until the benefits of such infections on DM are definitively understood.

### 4.2 Parasitic infection exacerbates diabetes

Furthermore, not all parasites have beneficial effects on the health of individuals with DM; indeed, harmful parasitic infections can worsen their condition. Chronic parasitic infections can lead to long-term complications, including organ damage, fibrosis, and metabolic disruptions (117). *T. gondii* has garnered considerable attention recently. Initial studies using mouse models demonstrated that acute T. gondii infection significantly swells and reduces the number of pancreatic Langerhans cells, indicating severe damage (114). In Australia, a study found prevalent T. gondii infections in Western Australia but no serological evidence linking these infections to T2D (118). Ashraf reported that the high incidence of chronic toxoplasmosis in Bangladesh might be associated with elevated secretion of the pro-inflammatory cytokine IL-12 (119). Salem et al. found that T. gondii infection could induce immune-metabolic responses; in the group with positive obesity and MetS, measurements such as trunk FM, HOMA-IR, chemerin, and IFN-γ were significantly higher than in the opposing group. There was a strong correlation between serum levels of chemerin and IFN-γ (P<0.001), which were positively associated with BMI, waist circumference (WC), total and trunk FM, HOMA-IR, cholesterol, and triglycerides, and negatively with high-density lipoprotein cholesterol (HDL-C) (120). Diabetic patients with positive anti-Toxoplasma IgG antibodies experienced a longer duration of DM compared to those with negative antibodies (7.14 ± 2.962 years versus 3.26 ± 1.583 years, P < 0.001) (121), suggesting that T. gondii may also be a factor in hypertension among T2D patients (122). Parasitic infections can also contribute to severe nutrient depletion, exacerbating metabolic disorders in diabetic patients. Chronic helminth infections may lead to anemia, protein deficiency, and micronutrient deficiencies, all of which can negatively impact glycemic control and overall metabolic function (14, 123). Yingklan noted that participants with *S. stercoralis* infection estimated glomerular filtration rate (eGFR) and higher levels of alanine aminotransferase (ALT) and urine albumin-to-creatinine ratio (UACR), indicating potential severe renal complications (124). Furthermore, co-infections with opportunistic pathogens pose a significant risk in patients receiving helminth therapy. Immunocompromised individuals, such as those with HIV/AIDS or undergoing immunosuppressive treatments, are more susceptible to severe opportunistic infections, including Cryptosporidium, cytomegalovirus (CMV), and bacterial superinfections (125, 126).

Parasitic therapy remains experimental. Although *S. stercoralis* infection negatively correlated with T2D in Northeast Thailand, infected individuals exhibited lower eGFR and higher ALT and UACR levels. In immunocompromised patients, inoculation with *S. stercoralis* could lead to co-infections with cytomegalovirus CMV, and worsening renal parameters associated with complications (127). The impact of parasites on gut microbiota composition also remains a key area of concern (128). While some helminths have been shown to promote beneficial microbiota shifts, others may increase gut permeability, leading to bacterial translocation and systemic inflammation, which can worsen metabolic diseases (129, 130). *Demodex folliculorum (D. folliculorum)*, a mite that causes demodicosis, is noted for facial damage and may be linked to altered immune responses in conditions such as T2D (104, 131). A 45-year-old woman with interstitial lung disease and localized scleroderma-related pulmonary arterial hypertension (PAH) began treatment with *Necator americanus (N. americanus)* as an alternative therapy. Despite treatment, her respiratory symptoms worsened after an increase in eosinophils and elevated IgE levels, with detected *S. stercoralis* IgG antibodies, highlighting the complex interplay between parasitic infections and chronic diseases (132).

Explaining Divergent Findings. Apparent inconsistencies across studies largely reflect (i) pathogen class—protozoa commonly drive Th1/Th17-polarized inflammation linked to β-cell stress, whereas helminths elicit Th2/Treg-leaning programs that promote tissue repair and metabolic tolerance; (ii) host context—genetic background, baseline inflammation, and metabolic comorbidities; (iii) infection features—stage (acute vs. chronic), parasite burden, and tissue tropism; and (iv) study design and endpoints (e.g., HOMA-IR, HbA1c, clamp, cytokines). Protozoan data are therefore treated as comparative context rather than counter-examples to helminthic therapy.

## 5 Control living therapeutic parasites

### 5.1 Improve safety and tolerance

Firstly, the selection of parasites with lower toxicity is paramount. For instance, among the various species of Plasmodium, *Plasmodium vivax (P. vivax)* demonstrates superior infection and replication abilities in the spleen and bone marrow compared to *Plasmodium falciparum (P. falciparum)*, which exhibits a relatively mild impact (133). Regarding therapeutic applications, *Taenia saginata (T. saginata)* is preferred over Trichuris suis ova (TSO), which is known to cause neurocysticercosis (134). Numerous clinical trials have highlighted the safety of certain parasites, including *T. solium* and Ancylostoma duodenale, as indicated in **Table 2**. However, the available data do not allow the determination of a safe dosage free of safety concerns. The group has therefore concluded that the safety of novel food remains unconfirmed (148). Additionally, by controlling the reproductive capabilities of parasites, both the population of parasites and the complications arising from their eggs can be reduced. For instance, manipulating hTNF-α to influence the development, metabolism, and egg-laying of parasites has significantly lessened the burden in mice infected with unisexual schistosomes (149, 150). Moreover, short interfering RNA (siRNA) targeting of SjGT and SjNCSTN can induce minor morphological changes in the testes of male nematodes, substantially diminishing their vitality and fertility (151). However, it is crucial to consider the variations in the biological traits and host interactions of parasites when implementing these strategies (152).

**Table 2:** The performance of helminths in clinical trials.

Helminth	Clinical trial phase	Target population	Results of marker changes		Conclusion	Ref.
			Increase	Decrease		
*TSO*	I	CD	FCP; CRP	/	• Safe and well-tolerated • No clinically significant changes in gastrointestinal symptoms and signs	(135)
*TSO*	II	CD	EOS; IgG; CAL; LF	CDAI; CRP	• Safe and well-tolerated • Did not significantly improve clinical symptoms to achieve clinical remission in CD	(78)
*TSO*	II	Allergic rhinitis	EOS; IgE; IgG; IgG4; IgA	/	• Acceptable safety in early-phase RCTs; efficacy not superior to placebo on primary endpoints. • No effect on allergic rhinitis	(77)
*TSO*	I	MS	IgG; IgE; Activated HLA-DR PB	/	• Acceptable safety in small early-phase cohorts; immunomodulatory signals observed. • The extent of changes in inter-individual T-cell responses and cell functions varies, leading to diverse overall clinical efficacy	(136)
*TSO*	I	MS	IL-4; EOS	IL-2	• Safety profile not specified • Moderated immunomodulatory effect on MS patients	(137)
*TSO*	I	ASD	IL-4; IL-5; IL-10; IL-13	/	• Only caused mild and non-serious side effects • Significantly improved ASD patients	(138)
*TSO*	I	Psoriasis	IFNγ; TNFα; IL-2; IL-4; IL-5; IgG; IFNγ^+^; IL4^+^; EOS	/	• Safety profile not specified • Significantly impacted the immune system of psoriasis patients	(139)
*TSO*	I	Obesity	EOS; Goblet cells	Hb; RBC; Weight	• Safe and well-tolerated	(140)
*TSO*	I	MS	IL-10; TGF-β; Treg cells; Th2 related cytokines; CD4^+^, CD8^+^ T cell; CD56^bright^ NK cell; EOS; BDNF; NGF	IL-12,IFN-γ,IL-2	• Safe and well-tolerated in relapsing-remitting multiple sclerosis • Effectively modulated the immune system and alleviated the condition of multiple sclerosis	(141)
*Hookworm*	I	MS	CD4^+^,CD25^high^ and CD127^neg^T cell; Treg; EOS	Treg	• Safe and well-tolerated in relapsing-remitting multiple sclerosis • Might effectively increase regulatory T cells, exerting a specific immunomodulatory effect, which may help alleviate the condition of multiple sclerosis	(90)
*N. americanus L3 larvae*	I	CeD	IL-2; EOS; IEL; CD4^+^, CD25^+^, CD3^+^ and CD8^+^ T cell; Foxp3^+^ cell	IFN-γ, IL-17A, Vh	• Safe and well-tolerated • Effectively suppressed Th1/Th17 inflammatory responses and had potential therapeutic effects on the pathology of celiac disease	(142, 143)
*N. americanus L3 larvae*	I	CD	IET; QoL	/	• Safe and well-tolerated, and the incidence of gluten-related adverse events was significantly reduced in the hookworm treatment group • Did not restore tolerance to continuous moderate gluten intake in celiac disease patients, but symptom scores improved after intermittent low gluten intake	(88)
*N. americanus L3 larvae*	I	Asthma	Bronchial responsiveness (DD); EOS	Lung function	• Safe and well-tolerated • Respiratory reactivity had a non-significant improvement	(144)
*N. americanus L3 larvae*	I	Allergic rhinoconjunctivitis	EOS	/	• Did not lead to a clinically significant worsening of respiratory reactivity, and overall, the infection was well-tolerated • Well-tolerated with no significant safety issues	(145)
*N. americanus L3 larvae*	I	Obesity	IL-4; IL-5; IL-13; IL-10; IL-17A; IL-2; IFNγ; TNF; IL-6; IL-1β	IL-12p70; IL-12p40; IL-18; IL-33	• Safe and well-tolerated • Provided a basis for potential mechanisms in limiting obesity and type 2 diabetes-related inflammation and metabolic cascades	(3)
*N. americanus L3 larvae*	I	Health	IFNγ; TNFα; IL-2; IL-4; IL-5; IgG; EOS	/	• More adverse events were reported than in the placebo group, but most were mild, indicating good overall tolerability • The vaccine induced a robust immune response to hookworm antigens and reduced fecal larvae output, showing potential protective efficacy	(146)
*N. americanus*	I	Health	IgG1; EOS; IL-8	Th2 Cytokines; IL-4; IL-1β	• Moderated safety and tolerability	(147)
*N. americanus*	II	CeD	EOS; T cells producing IFN-γ; Duodenal IEL count; Marsh score (Mucosal damage)	Hb; Vh/Cd	• Safe and well-tolerated	(143)
*N. americanus* *L3 larvae*	Ib	Adults at risk of T2D (n=40)	EOS	HOMA-IR**;** FBG (12 months)**;** Body mass (24 months; L3–20 arm)	• Acceptable safety with mainly mild–moderate GI adverse events; feasibility signal on metabolic endpoints; Phase II/III warranted.	(3, 76)

TSO, Trichuris suis Ova; CD, Crohn’s Disease; FCP, Fecal Calprotectin; CRP, C-reactive Protein; CAL, Calprotectin; LF, Lactoferrin; CDAI, Crohn's Disease Activity Index; MS, Multiple Sclerosis; HLA-DR PB, HLA-DR High-quality Membrane Plasmablasts; ASD, Autism Spectrum Disorder; Hb, Hemoglobin; RBC, Red Blood Cell; BDNF, Brain-Derived Neurotrophic Factor; NGF, Nerve Growth Factor; CeD, Celiac Disease; VH/CD, Duodenal Villus Height/crypt Depth; IET, Intraepithelial T; QoL, Quality of Life Rate; DD Dose-Dependent. Protozoan trials are shown separately in **Supplementary Table S1**. Comparative non-helminth infection models, including controlled human malaria infection and sporozoite immunization trials, have been reported in several studies (160–163).

We can also reduce the virulence of parasites through artificial methods. For instance, adding gentamicin during *in vitro* culture can maintain the long-term low virulence of wild-type parasites (153). Similarly, the application of genetic engineering techniques, such as irradiation or gene editing, can effectively lessen the virulence of parasites (154, 155). These methods have demonstrated potential in experimental studies with hookworms, aimed at treating conditions such as asthma (155), cancer (156), ulcerative colitis (157), and anemia (158). The rapid advancements in bioengineering have ushered in next-generation CRISPR gene editing tools like CRISPR-Cas13 (159), enabling the modification of parasites to attain specific traits such as reduced life cycles, inhibited proliferation, or increased drug sensitivity. RNA interference techniques have been utilized to silence target mRNA transcription, aiding in elucidating gene functions in nematodes, with proven efficacy in Caenorhabditis elegans. The CRISPR/Cas9 system facilitates the knockout or deletion of specific genes in parasitic worms, though the potential for gene insertion remains largely unexplored (164). CRISPR technologies and their derivatives are poised to advance, potentially leading to novel methods for managing protozoan parasites (165).

Gene editing has been shown to effectively reduce the virulence of parasitic organisms across various species by targeting specific genes associated with pathogenicity. In the liver fluke *O. viverrini*, CRISPR/Cas9-mediated knockout of the *OV-GRN-1* gene has been demonstrated to reduce pathological effects, as evidenced by decreased biliary hyperplasia and fibrosis in infected hosts. This finding underscores the role of *OV-GRN-1* in virulence during opisthorchiasis (166). Similarly, in *Toxoplasma gondii*, CRISPR-Cas9 deletion of the *wx2* gene significantly inhibits parasite growth and replication *in vitro* and leads to a reduction in virulence *in vivo*. This effect is primarily mediated through modulation of the host immune response via the Th1 and Th17 pathways (167). In malaria parasites, zinc-finger nucleases have been utilized to induce double-strand breaks, resulting in developmentally arrested attenuated strains. This approach presents a promising strategy for vaccine development (168). Additionally, the CRISPR-Cas9 system has been applied to *Plasmodium falciparum* to enable rapid gene deletion and nucleotide substitution, facilitating gene function studies and the identification of novel drug targets (169). The application of CRISPR in parasitology extends to *Eimeria tenella*, where gene editing has been used to investigate gene function and identify essential genes for parasite development and survival. These studies provide valuable insights into potential therapeutic targets (170). Finally, in the nematode *N. brasiliensis*, a novel method utilizing extracellular vesicles for CRISPR/Cas9 component delivery has successfully disrupted secreted DNase II. This disruption resulted in reduced gene expression and demonstrated the feasibility of genetic manipulation in parasitic nematodes (171). Research also indicates that CRISPR/Cas9 can enhance the safety of parasitic eggs, particularly those of schistosomes, by reducing their pathogenicity (172). Experimental results show that eliminating the omega-1 protein significantly reduces the size of pulmonary granulomas in mice (173). This protein is a soluble egg antigen known to damage tissues and modulate the Th2 immune response (174), thereby attenuating parasite toxicity (173). CRISPR/Cas9 can also alter the acetylcholinesterase (AChE) gene in parasites, diminishing their activity and eliciting a Th2 immune response, which is essential for enhancing host defenses against parasites and potentially reducing resistance to atovaquone (175, 176). Furthermore, employing electroporation has proven to improve the efficiency of CRISPR/Cas9 gene editing (177), and studies have demonstrated that Cas12a offers superior gene editing capabilities compared to Cas9 (178). These advancements are crucial for improving the efficiency of clinical products related to parasites. While gene editing efficiencies are low, exploring new delivery mechanisms and markers could optimize these methods and foster further technological developments (179).

### 5.2 Real-time monitoring of therapeutic efficacy

With modern medicine and information technology advancements, research into parasitic therapy is evolving. Rapid developments in intelligent detection technologies are now applied to monitoring DM-related markers and increasingly detecting parasites, revealing intricate interactions and potential therapeutic mechanisms between parasites and their hosts. For instance, glucose-responsive nanoparticles facilitate rapid and extended self-regulation of insulin delivery (180, 181). Similarly, smartphone-assisted microfluidic chemical analyzers use image-based colorimetry for comprehensive monitoring of DM and hyperlipidemia, offering real-time tracking of dynamic patient information (182).

Drawing on the concepts used in glucose-responsive materials and intelligent detection technologies, it is possible to continuously monitor the effectiveness of parasitic formulations and the patient’s status, enhancing the safety and efficacy of parasitic therapy. Patients receiving helminthic therapy are often subjected to regular medical monitoring, such as PET/MRI scans (133), to evaluate morphological and metabolic changes in organs affected by infection, ensuring that parasite levels remain safe. The incorporation of state-of-the-art monitoring technologies like the Helminth Egg Automatic Detector (HEAD) (183), Kato-Katz method (184), Mini-FLOTAC technique (185), qPCR (186), and Ov-RPA-CRISPR/Cas12a system (187) enables regular medical examinations during treatment, monitoring parasite numbers and activity to maintain control of the infection. The advantages and disadvantages of different new monitoring technologies are summarized in **Table 3**.

**Table 3:** Comparison of the advantages and disadvantages of new monitoring technologies.

Technology	Advantages	Benefits	Applications	Limitations	Cost	Clinical value	Ref.
Glucose-responsive nanoparticles	• Rapid blood glucose response • Long-term self-regulated insulin release	• Stable glucose control • Reduced risk of hypoglycemia	• Insulin regulation for diabetes • Dynamic blood sugar monitoring	• Complex manufacturing • Limited market availability	High	• Precise glucose management • Enhanced long-term metabolic health	(188–190)
Smartphone-assisted microfluidics	• Portable, easy home use • Colorimetric results are user-friendly	• Boosts treatment adherence • Enables remote healthcare access	• Home monitoring for diabetes • Remote medical support	• Relies on smartphone & network availability • External light impacts precision	Medium	• Real-time patient monitoring • Suitable for primary care diagnostics	(191, 192)
PET/MRI scans	• High-resolution imaging • Tracks parasite infection and therapy progress	• Monitors organ and metabolic changes • Provides precise therapeutic feedback	• High-risk patient monitoring • Post-treatment assessments	• High cost, specialized equipment needed • Not suitable for resource-limited settings	Very High	• Tracks parasite-related organ damage • Ensures infection control	(193)
Helminth Egg Automatic Detector	• Automated parasite egg detection • High-speed screening	• Rapid, accurate treatment evaluation • Reduces lab workload	• Monitoring parasite burden • Treatment dose adjustments	• Limited sensitivity to specific eggs • High-tech complexity	Medium-High	• Accurate parasite burden data • Adjusts therapy based on precise data	(194, 195)
Kato-Katz method	• Low-cost, widely accessible	• Suitable for low-resource areas	• Initial screening & monitoring	• Low sensitivity for mild infections	Low	• Affordable diagnostic option	(196, 197)
qPCR (Quantitative PCR)	• High sensitivity for low parasite DNA levels • Multi-parasite detection	• Early detection & therapy monitoring	• Genetic testing for infections	• High cost and technical expertise required • High-quality samples needed	High	• Essential for early, accurate diagnosis • Supports precision medicine	(198)
*Ov-RPA-*CRISPR/Cas12a system	• High specificity and sensitivity • Quantifies parasite DNA/proteins	• Early infection detection	• Research & infection monitoring	• Complex and time-consuming sample steps	High	• Accurate diagnosis for infections • Personalized treatment & monitoring	(199, 200)

Furthermore, leveraging big data to explore new protein molecules, analyze parasite-human interactions, and acquire proteomics data from *H. contortus* through bioinformatics (201) are critical for harnessing parasites for human benefit. We also introduce a convolutional neural network (CNN) for the classification and automated detection of *O. viverrini* eggs from digital images (202). Digital imaging systems for identifying and quantifying pathogenic helminth eggs (203), together with the Helminth Egg Analysis Platform (HEAP) — an open platform integrating deep learning architecture for microscopically identifying and quantifying helminth eggs (204) — represent the forefront of this research area (**Figure 3**).

In conclusion, by integrating intelligent nanomaterial delivery systems with sophisticated monitoring technologies such as image-based colorimetry, microfluidic chemical analysis, and automatic helminth egg detection, we can achieve real-time surveillance of biochemical markers in diabetic patients and treatment outcomes in those undergoing parasitic therapy. This improves the precision and timeliness of treatments and opens new avenues for future medical interventions.

### 5.3 Anthelmintic drugs targeting glucose metabolism

In cases where patients do not achieve the anticipated therapeutic effects during parasite therapy or experience severe complications or immune responses, the immediate administration of anthelmintic drugs represents the most direct strategy to prevent the worsening of adverse reactions. Currently, pyrroloquinoline class drugs are the first choice for clinical antiparasitic treatments (205). However, due to growing concerns about resistance to these drugs (206), new anthelmintics targeting alternative biological pathways have been introduced. Notably, drugs targeting the glycometabolic pathways of parasites offer significant advantages for treating patients with T2D.

Parasites rely heavily on glucose as an energy source. Their metabolic competition for glucose results in enhanced uptake and metabolism by host cells to offset energy deficits (207). Research indicates that individuals with DM are more prone to parasitic infections, possibly due to higher glucose levels in the blood, which can directly influence blood sugar changes, particularly affecting men more significantly (208). Schistosomes, highly dependent on glucose for survival and reproduction, suffer in their ability to live and reproduce when glucose availability is inhibited (209). Targeted pharmaceutical research has identified vital glucose transport proteins (SGTPs) and enzymes within the glycolytic pathway in *S. mansoni* and *S. japonicum* as potential drug targets. Four compounds—pyrroquinoline, licochalcone A, licarin, and harmonine—have effectively inhibited SGTP4 (210), which is crucial for extracting glucose from the host’s bloodstream. Recent studies suggest that protein kinase B (Akt) is vital for SGTP4 expression (211), and inhibiting Akt reduces glucose uptake by the parasites (212). Moreover, deficiencies in glucose-6-phosphate dehydrogenase (G6PD) and pyruvate kinase (PK) in Plasmodium have been shown to protect against the severe effects of malaria, underscoring the enzymes’ critical role in the parasite’s lifecycle, thereby making PfGluPho a promising target for antimalarial drugs (213). Additionally, the fusion enzyme G6PD::6PGL in Giardia lamblia, with its increased glycometabolic efficiency and structural differences from human G6PD, represents a novel target for developing anti-Giardia medications (214, 215).

**Figure 5:**
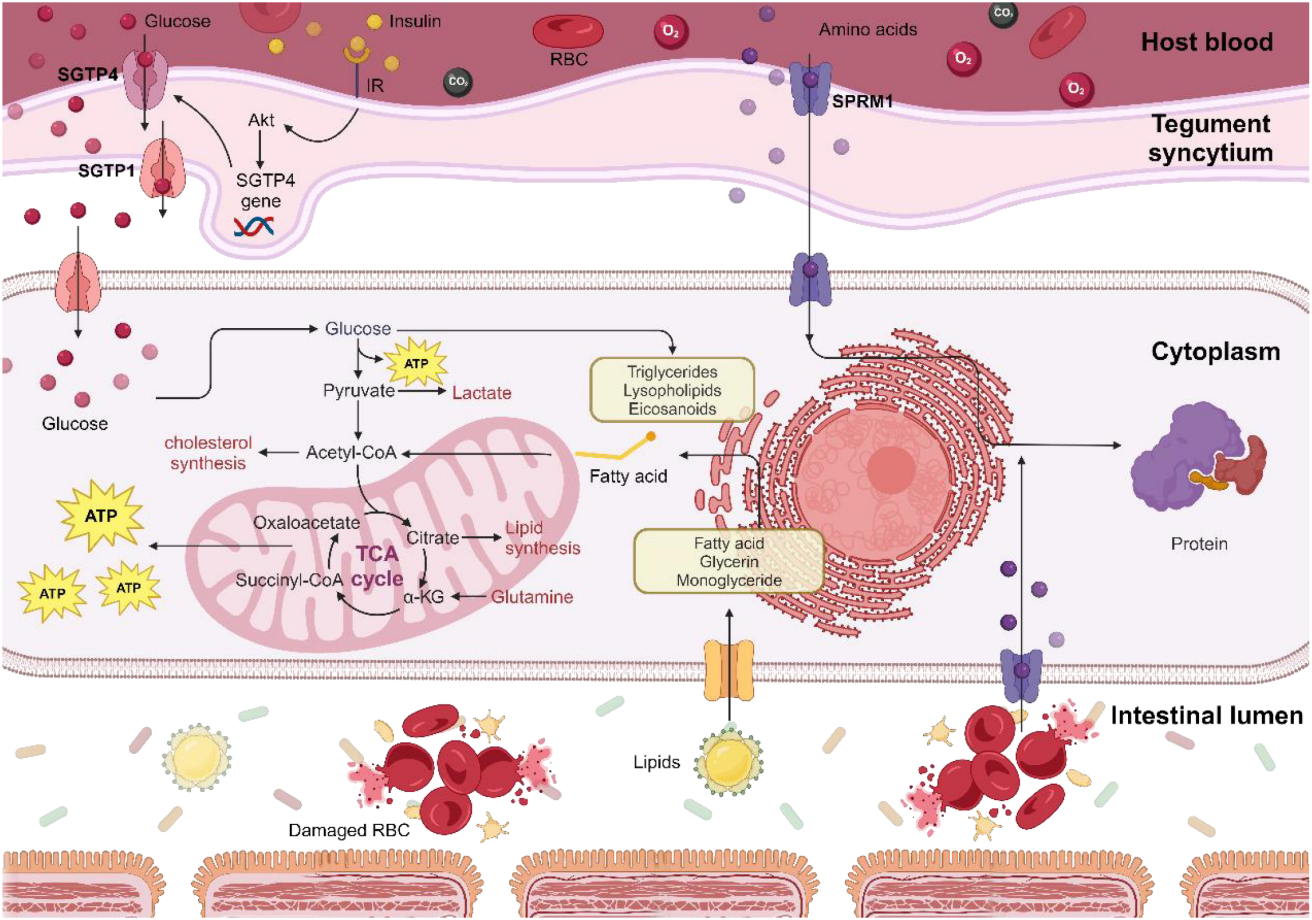
Mechanisms of energy metabolism in schistosomes. Schistosomes exploit host-derived glucose, lipids, proteins, and amino acids for metabolic energy production and as precursors for synthesizing their essential biomolecules, which are crucial for their growth, reproduction, and egg-laying activities. Consequently, targeting glucose uptake by schistosomes could offer heightened therapeutic sensitivity in patients with T2D.

By targeting the glycometabolic processes essential for parasite survival and reproduction, we can precisely regulate their population within the host and mitigate their impact on host glucose metabolism (**Figure 5**). Furthermore, the inhibition of parasite-specific insulin receptors and human TNF-α has significantly decreased parasite glucose consumption, thus mitigating the adverse effects of parasite therapy (149, 216).

### 5.4 The therapeutic application of molecules derived from helminths

Parasitic infections typically lead to pathological conditions; however, the therapeutic application of helminth-derived molecules in DM treatment has gained increasing interest in recent years. These molecules regulate the host immune response through multiple mechanisms and have shown potential therapeutic effects in both T1D and T2D.

Recent studies have highlighted the significant immunomodulatory properties of *Fasciola hepatica* secretions, which have been shown to prevent T1D in NOD mice. These secretions induce M2 macrophages and regulatory T cells, thereby suppressing pancreatic inflammation, reducing autoimmune destruction, and effectively halting DM progression (217). Similarly, the *Fasciola hepatica*-derived protein FhHDM-1 has demonstrated the ability to activate the PI3K/Akt signaling pathway and preserve β-cell mass in NOD mice, protecting against cytokine-induced apoptosis and enhancing insulin secretion (102). In addition to trematode-derived molecules, nematode-derived proteins from *Wuchereria bancrofti* (*W. bancrofti*) and *Brugia malayi* (*B. malayi*) have exhibited promising therapeutic potential in diabetic mouse models. These proteins lower blood glucose levels and reduce islet inflammation by inhibiting pro-inflammatory cytokines such as TNF-α and IFN-γ, while simultaneously promoting anti-inflammatory cytokines, including IL-4, IL-5, and IL-10 (218). This immunoregulatory mechanism further supports the potential of helminth-derived molecules in DM treatment. Beyond their effects on T1D, helminth infections have also shown benefits in T2D models. For instance, *S. mansoni* infection has been found to enhance Th2-type immune responses, leading to improved insulin sensitivity in obese mice and reduced inflammation in adipose tissue. This suggests that helminth infections may alleviate metabolic disturbances associated with T2D by modulating systemic immune responses (6). Similarly, *S. mansoni*-derived soluble egg antigens (SEA) and ω1 have been shown to enhance metabolic homeostasis and insulin sensitivity through immune modulation, particularly by promoting Th2 cells, eosinophils, and M2 macrophages in adipose tissue (208, 209). Additional research has demonstrated that antigens from *Litomosoides sigmodontis* (*L. sigmodontis*) can enhance the efficacy of insulin-specific therapy. When combined with insulin treatment in NOD mice, these antigens effectively prevent the onset of T1D, even in the presence of early pancreatic inflammation (P > 0.05) (219). Likewise, hookworm-derived secretions have exhibited potential in improving T2D-related metabolic disorders. Studies indicate that these secretions enhance glucose tolerance in diabetic mouse models and reduce systemic inflammation by modulating gut microbiota and local immune responses, ultimately improving insulin sensitivity (220). Furthermore, excretory-secretory products (ESPs) from *N. brasiliensis*, including those from adult and third-stage larvae, have been shown to significantly lower fasting blood glucose levels and improve glucose metabolism in HFD-fed C57BL/6 mice. These effects are accompanied by immune modulation, characterized by increased eosinophil and IL-5 levels and decreased IL-6 levels in adipose tissue (207). These results highlight the therapeutic potential of worm-derived molecules, which modulate both metabolic and immune pathways, offering new strategies for managing DM and related metabolic conditions.

### 5.5 Challenges in parasite engineering and monitoring

Delivery and editing efficiency. Genome engineering in multicellular helminths remains limited by stage-specific physical barriers (egg shell, cuticle) and low transformation/editing rates across life stages. Proof-of-concept CRISPR/Cas9 editing in Schistosoma mansoni and the recent generation of stable transgenic lines in Strongyloides stercoralis demonstrate feasibility, yet frequent mosaicism and limited heritability still complicate phenotype attribution and scale-up, indicating platform-level optimization is required before translation (173, 221).

Off-target risk and validation. Translational applications require genome-wide, unbiased off-target surveillance beyond in silico predictions. CHANGE-seq provides sensitive and scalable maps of nuclease activity and should be coupled with amplicon deep sequencing or whole-genome sequencing across relevant life stages to minimize false attribution of engineered phenotypes (222).

Monitoring readouts and clinically meaningful endpoints. For parasite burden, circulating antigen assays (e.g., CCA/CAA) outperform microscopy at low intensities in schistosomiasis and are increasingly recommended for surveillance and clinical evaluations, although implementation remains heterogeneous across settings. For host metabolism, helminth–metabolism trials should pre-specify HOMA-IR, fasting plasma glucose, HbA1c and—when feasible—CGM-derived time-in-range (TIR) per contemporary diabetes standards to enable cross-study comparability (223, 224).

Regulatory and biosafety expectations. Clinical development of live or engineered helminths should meet LBP-like Chemistry, Manufacturing, and Controls (CMC) expectations (identity/potency, sterility, genomic stability) under U.S. Food and Drug Administration (FDA) guidance and comply with the updated National Institutes of Health (NIH) Guidelines for recombinant/synthetic nucleic acid research (Institutional Biosafety Committee (IBC) oversight; appropriate containment for gene-drive-related constructs), with jurisdiction-specific alignment. Protocols must include rescue therapy, environmental containment/kill-switch plans, and post-trial surveillance.

## 6 Proposal for clinical decision-making

### 6.1 Risk assessment

In the application of parasitic therapy for DM treatment, establishing appropriate patient selection criteria is essential for ensuring both its adaptability and safety. To address this need, we have developed a comprehensive risk assessment framework that integrates host factors, parasitic factors, treatment parameters, and environmental considerations to evaluate the suitability and potential risks of parasitic therapy for individual patients. (*i*) Host Factors: These include DM type (225, 226), glycated hemoglobin (HbA1c) levels (227), homeostatic model assessment of insulin resistance (HOMA-IR) (228), baseline inflammation levels (229), and immune status (230). These indicators provide critical insights into the patient’s metabolic condition and inflammatory response, serving as key criteria for determining their eligibility for parasitic therapy. (*ii*) Parasitic Factors: Given that different parasite species and their life cycle stages exert varying effects on the host, we assess their pathogenicity and developmental stage to determine the most appropriate and safe parasite type for therapeutic use (231–233). (*iii*) Treatment Parameters: Factors such as treatment dosage and frequency are tailored to each patient based on prior risk assessment results, optimizing therapeutic efficacy while minimizing potential adverse effects (234, 235). (*iv*) Environmental Factors: Considerations such as hygiene conditions are crucial in mitigating the risk of infections that may occur during treatment (82, 236). The risk scoring table and risk classification are shown in **Table 4a** and **Table 4b**, respectively.

**Table 4a:** Comprehensive risk assessment scoring criteria.

Category	Parameter	Scoring Criteria	Ref.
Host Factors	1. Diabetes Type	T1DM: +2 points; T2DM: 0 points; Gestational Diabetes: +3 points	(225, 226)
	2. HbA1c (%)	<7%: 0 points; 7-8.5%: +1 point; >8.5%: +3 points	(227)
	3. HOMA-IR	≤2.5: 0 points; 2.5-4.0: +1 point; >4.0: +2 points	(228)
	4. Baseline Inflammation	≤3 (hs-CRP, mg/L): 0 points; 3-10: +1 point; >10: +2 points	(229)
	5. Immune Status	Normal immune function: 0 points; Immunosuppressant use: +3 points	(230)
Parasite Factors	6. Pathogenicity of Species	Non-pathogenic (e.g., Trichuris suis): 0 points; Moderately pathogenic (hookworms): +1 point; Highly pathogenic (schistosomes): +3 points	(231, 232)
	7. Parasite Lifecycle Stage	Eggs/Larvae: 0 points; Adults (capable of reproduction): +2 points	(233)
Therapeutic Factors	8. Dosage (larvae/session)	≤10: 0 points; 10-50: +1 point; >50: +3 points	(234)
	9. Treatment Frequency	Single dose: 0 points; Quarterly: +1 point; Monthly: +2 points	(235)
External Factors	10. Hygiene Conditions	High (sterile environment): 0 points; Moderate (home care): +1 point; Low (open environment): +3 points	(82, 236)

**Table 4b:** Risk stratification and management recommendations.

Total risk score	Risk level	Management recommendations	Ref.
0-4	Low Risk	Standard treatment, monitor HbA1c and stool ova every 3 months	(232, 237)
5-8	Moderate Risk	Halve the dosage, monitor HbA1c, IL-6, liver/kidney function, and blood biochemistry monthly	(229, 234, 236)
≥9	High Risk	Contraindicated; requires multidisciplinary consultation (endocrinology and infectious disease specialists)	(82, 231, 238)

Based on these variables, we have developed a quantitative risk scoring system to systematically assess each patient’s overall risk profile. Patients are categorized into three risk levels—low, medium, or high—each corresponding to specific treatment recommendations. This stratified approach enables a more personalized treatment strategy while maintaining safety and efficacy. By implementing this risk assessment framework, we provide clinicians with a structured decision-making tool to determine patient eligibility for parasitic therapy, identify those requiring specialized treatment strategies, and recognize cases where the therapy should be avoided altogether. This systematic approach not only enhances the precision and personalization of treatment but also improves patient safety and overall treatment satisfaction.

### 6.2 Dosage and duration of treatment

The appropriate selection of parasite species, dosing regimen, and treatment duration is critical for ensuring the safety and efficacy of helminth therapy in patients with T2D. Based on current clinical trials and experimental studies, *N. americanus* (American hookworm) and *Trichuris suis* ova (TSO, pig whipworm eggs) are the most extensively studied helminths for T2D treatment. The typical dosages are as follows.

*N. americanus*: In a randomized, double-blind, placebo-controlled Phase Ib trial of 40 adults at risk of type 2 diabetes, a single percutaneous inoculation of 20 or 40 L3 larvae was evaluated; at 12 months, both doses showed signals of improvement in HOMA-IR and fasting glucose, and the 20-larvae arm exhibited body mass reduction at 24 months. Adverse events were predominantly mild–moderate gastrointestinal symptoms with acceptable overall tolerability; dose, follow-up schedule, and predefined metabolic endpoints are detailed in the published protocol. Accordingly, 20 or 40 L3 is recommended as starting doses in clinical studies with rigorous metabolic and safety monitoring (3, 76).

TSO: Clinical studies recommend an initial dose of 2,500 eggs, administered orally every two weeks, with potential escalation to 7,500 eggs based on patient tolerance and therapeutic response. The standard treatment duration is 12–24 weeks, during which immune and metabolic parameters—such as cytokine levels (IL-10, TNF-α), homeostatic model assessment of insulin resistance (HOMA-IR), and fasting blood glucose—should be closely monitored (135, 239).

Dose titration is essential for minimizing adverse effects and optimizing therapeutic outcomes. Factors such as body weight, baseline glycemic control, and especially HbA1c should be considered (**Table 5**). Patients with lower body weight or elevated baseline inflammatory markers may require lower initial doses to prevent excessive immune activation (245, 246). Routine assessment of key biomarkers—including CRP, eosinophil count, and gastrointestinal symptoms—is advisable to guide gradual dose escalation or reduction (246, 247). The duration of therapy should be tailored to individual patient needs and treatment goals. For patients with well-controlled blood glucose, a shorter treatment course (e.g., 12 weeks) may suffice, whereas those with poor glycemic control or severe insulin resistance may require an extended course of up to 24 weeks (248). Long-term follow-up is essential for evaluating the durability of therapeutic benefits and detecting delayed adverse effects, such as gut microbiota dysbiosis or residual parasite burden (249).

**Table 5:** HbA1c changes and treatment adjustment recommendations.

Response type	HbA1c change	Dose adjustment recommendation	Notes	Ref.
Good Response	Decrease >1%, no inflammation worsening	Maintain current dose	• Continuously monitor treatment stability. • Ensure no adverse effects or inflammation worsening.	(227, 240, 241)
Partial Response	Decrease 0.5-1%	Increase dose to the maximum safe threshold	• If the desired outcome is not achieved, cautiously increase the dose. • Monitor patient safety closely.	(236, 242)
No Response	Change <0.5%	Switch to helminth-derived molecules or combine with immunomodulators	• Consider modifying the treatment strategy. • Enhance efficacy by integrating other immunoregulatory methods.	(15, 74, 232, 243, 244)

### 6.3 Monitoring during the treatment period

In the application of parasitic therapy, continuous and precise patient monitoring is of primary importance (250). Advances in modern medicine and information technology, particularly the integration of intelligent detection systems and nanomaterials, have the potential to enhance the monitoring capabilities of parasitic therapy for DM treatment. The proposed monitoring protocol is designed to comprehensively assess therapeutic outcomes and their physiological effects on patients, ensuring both safety and efficacy. Once treatment begins, the monitoring plan should include routine blood glucose and HbA1c tests every three months, which help evaluate DM management and assess the therapy’s impact on insulin resistance (251, 252). Additionally, inflammatory markers and lipid levels should be assessed every six months, as this is crucial for monitoring chronic inflammation and cardiovascular health (253). These periodic evaluations facilitate timely adjustments to the treatment regimen, ensuring optimal therapeutic outcomes (254). Following the completion of treatment, annual follow-up examinations will focus on the long-term control of DM and changes in gut microbiota—key factors in assessing the sustained efficacy and broader physiological effects of parasitic therapy (3). This comprehensive monitoring protocol provides physicians with a complete dataset on treatment progression, allowing for more precise adjustments to personalized treatment plans (see detailed **Table 5**).

### 6.4 Ethical and regulatory considerations

Clinical translation of helminth-based interventions raises substantial ethical and regulatory challenges.

Ethical oversight and informed consent. Participants must be provided with clear and comprehensive information about potential risks (e.g., acute infection, immunopathology, allergic reactions), uncertain benefits, and long-term unknowns, and they must retain the right to withdraw at any time. Vulnerable groups such as pregnant individuals, children, and immunocompromised persons require special protection. Long-term follow-up (≥12–24 months where feasible) is ethically essential to capture delayed or chronic adverse effects (255).

Regulatory classification and quality control. Live helminth therapies are likely to be classified as Live Biotherapeutic Products (LBPs) or biologics under authorities such as the FDA (US) and European Medicines Agency (EMA) (EU). Regulatory guidance emphasizes stringent requirements for identity, purity, potency, viability, and stability, with well-defined Chemistry, Manufacturing, and Controls (CMC) procedures to ensure batch-to-batch consistency, traceability, and freedom from adventitious agents (256, 257).

Safety monitoring and risk mitigation. Clinical protocols should guarantee immediate availability of rescue anthelmintics to terminate infection in case of adverse events. Independent Data Safety Monitoring Board (DSMB) oversight and standardized adverse event (AE) reporting are required. Predefined stopping rules should be implemented to halt trials in response to serious safety concerns (256).

Environmental and biosafety considerations. Trials involving genetically modified helminths or non-endemic species must include robust biosafety and containment strategies to prevent accidental release. Risk assessments should evaluate persistence, transmissibility, and ecological consequences.

Alternatives and translational pathways. Given the ethical and regulatory complexity of live infection, many groups advocate prioritizing helminth-derived molecules or excretory/secretory products (ESPs) as lower-risk alternatives. These may reproduce immunomodulatory benefits while avoiding risks of live organism administration (258, 259).

## 7 Conclusion

Helminthic therapy presents a promising yet complex approach for DM management due to its immunomodulatory and metabolic regulatory effects. However, its broader clinical application remains constrained by significant challenges, including infection risks, patient-specific variability in therapeutic response, and the need for long-term safety data. Addressing these concerns requires a more refined understanding of host-parasite interactions, strategic risk mitigation, and the integration of intelligent monitoring technologies to optimize patient outcomes.

Future research should focus on elucidating the precise molecular and immunological mechanisms underlying the therapeutic effects of helminths. While studies have demonstrated improvements in insulin sensitivity and metabolic homeostasis, the specific pathways through which helminths modulate glucose metabolism remain insufficiently explored (260). Additionally, there is a pressing need for longitudinal clinical trials to assess the long-term safety and efficacy of helminthic therapy in diverse patient populations. Large-scale studies incorporating real-world data will be essential to determine the sustainability of treatment benefits and to identify potential delayed adverse effects (261, 262). Furthermore, epidemiological investigations into the inverse correlation between helminthic infections and DM prevalence across different geographic regions could provide valuable insights into population-level metabolic trends (263). From a clinical perspective, developing practical guidelines for patient selection and risk stratification is crucial to ensuring treatment safety and efficacy. Not all diabetic patients are suitable candidates for helminthic therapy, and those with compromised immune function or chronic infections may be at greater risk for adverse effects (264). A structured framework should be established to categorize patients based on their metabolic profile, immune response, and infection susceptibility, allowing for more personalized and controlled therapeutic interventions (265). Treatment regimens should also be optimized to determine the most effective dosing strategies and duration of therapy. The integration of helminthic therapy with conventional anti-diabetic treatments, such as metformin or Glucagon-Like Peptide-1 (GLP-1) receptor agonists, may enhance therapeutic outcomes while mitigating risks associated with live parasite exposure (14). Advancements in monitoring technologies will play a pivotal role in improving the safety and precision of helminthic therapy. Real-time tracking of treatment responses using AI-driven biomarker analysis, wearable biosensors, and digital health tools could provide continuous feedback on patient health status (266). Machine learning algorithms analyzing individualized patient data may further enable predictive modeling, allowing clinicians to tailor treatment regimens dynamically (267). Additionally, developing genetically modified helminths with reduced pathogenicity and reproductive potential may minimize adverse effects while preserving immunotherapeutic benefits. Risk mitigation strategies must also incorporate contingency measures for potential complications (268). Patients undergoing helminthic therapy should have access to immediate anthelmintic interventions if unexpected side effects arise. Moreover, targeted anthelmintic drugs that selectively suppress helminth survival without disrupting their beneficial immune effects could improve treatment safety (269, 270). In addition, clinical translation will also require strict adherence to ethical and regulatory standards, including frameworks governing live biotherapeutic products, to ensure patient safety, infection control, and public acceptance (256, 257).

Despite these challenges, helminthic therapy remains a compelling avenue for future metabolic disease management. With robust ethical and regulatory safeguards in place, together with biotechnological and personalized strategies, this therapy has the potential to evolve into a safe, effective, and accessible therapeutic option. Continued interdisciplinary research and innovation will be key to unlocking the full therapeutic potential of helminths and translating their immunomodulatory properties into viable clinical applications. Overall, therapeutic signals appear species-specific and context-dependent, emphasizing careful stratification by pathogen class, host background, and infection dynamics.
