# Supplementary material for: Clinical challenges and technological breakthroughs in helminthic therapy for diabetes
Source: Front Immunol. 2025 Nov 5;16:1642707. doi: 10.3389/fimmu.2025.1642707 (PMC12626982; doi:10.3389/fimmu.2025.1642707)
Supplement: Supplementary file 1 [file Table1.docx]

Supplementary Table S1. Non-helminth parasitic challenge/immunization models (comparative context): CHMI/IBSM/CPS trials and endpoints.

| **Organism / Exposure model** | **Clinical Trial Phase** | **Target Population** | **Results of Marker Changes** | | **Conclusion** | **Ref.** |
| --- | --- | --- | --- | --- | --- | --- |
|  |  |  | **Increase** | **Decrease** |  |  |
| *P. falciparum* | I | CHMI | / | PMR | - Safe and well-tolerated |  |
| *P. falciparum* | I | CHMI | IgG | Antibody Titers Against Sporozoite Extracts of NF54, NF135, and NF175 | - The safety and tolerability of these CPS regimens are poor, with many participants requiring medical intervention, indicating relatively low safety and tolerability | [1] |
| *P. vivax* | I | IBSM | ALT; AST; TBIL | / | - Safe and well-tolerated | [2] |
| *P. falciparum* | I | CHMI | IgG; Anti-CSP Titer | ISSR | - Good safety and tolerability, with no blood-stage infections observed in any subjects | [3] |

Abbreviations: CHMI, Human Malaria Infection; PMR, Parasite Multiplication Rate; CPS, Chemoprophylaxis; IBSM Induced Blood Stage Malaria; ALT, Alanine Aminotransferase; AST, Aspartate Aminotransferase; TBIL, Total Bilirubin; ISSR, Immune serum suppression.

1. van der Boor, S.C., et al., *Whole sporozoite immunization with Plasmodium falciparum strain NF135 in a randomized trial.* BMC medicine, 2023. **21**(1): p. 137.

2. Griffin, P., et al., *Safety and reproducibility of a clinical trial system using induced blood stage Plasmodium vivax infection and its potential as a model to evaluate malaria transmission.* PLoS neglected tropical diseases, 2016. **10**(12): p. e0005139.

3. Kublin, J.G., et al., *Complete attenuation of genetically engineered Plasmodium falciparum sporozoites in human subjects.* Science translational medicine, 2017. **9**(371): p. eaad9099.
